# The Army Medical Department

**Published:** 1898-04-02

**Authors:** 


### The Army Medical Department.

The medical officers of the army are much indebted to Dr. Farquharson and Sir William Priestley for the very decided way in which the grievances of the medical department were brought before the House of Commons on Friday; and it was remarkable that the customary defenders of the existing state of things were conspicuous by their absence from the debate. In reply to Dr. Farquharson, Mr. Powell Williams said that important changes in the department had been decided upon, that the medical branch of the service would be formed into what would be called the Army Medical Corps, that the existing and absurd double titles would be abolished, and that the officers would be simply colonel, major, captain, or lieutenant in the new corps. The prefix "Royal," which had been urgently solicited by the department, could only be granted by the consent of Her Majesty; and nothing was stated which could throw any light on the probability of that consent being accorded. Some difficulty existed with regard to the title of major-general, the possession of which would imply a general command; but this, no doubt, would admit of accommodation. Mr. Williams expressed the desire of the War Office to establish a complete understanding with the medical profession, and to restore the flow of candidates which has of late years been so seriously interrupted and disturbed. Dr. Farquharson was gratified by the tenor of the reply, and thought that the administration of the present Secretary of State for War would be made memorable by his having succeeded in bringing back peace and contentment to the department; but Captain Sinclair, while hoping for the best, said that it was impossible not to feel misgivings, and for this attitude of mind the speech of Sir William Priestley afforded ample material. Sir William repeated the familiar complaint that the present conditions of service of the medical officers have produced a social separation between them and the combatant officers, and he illustrated this by a description of the different positions occupied by two brothers, one of whom is in the combatant, while the other is in the medical branch. There can be no question that such a state of things has to some extent been called into existence, and it is likely enough that it will only gradually be removed. It is due partly to the severance of the bond which once existed between the regimental doctor and his brother officers, and partly to the fact that the blundering of the War Office in connexion with the whole subject, has deprived the Army of many of the class of men who formerly entered it, and has replaced them by others who, even if of equal professional competency, are not so constantly derived from the same social stratum as that by which combatant officers are supplied. The War Office thought that doctors were plentiful; and so, as a matter of cheeseparing, they dealt with them in such a manner as to destroy the attractiveness of the service.

A question of greater importance than this, however, arises from the fact that we may possibly be on the eve of a war of which no one can foresee the magnitude or the duration, and for which, whether it occur or not, it is imperatively necessary that the country, and the military and naval services, should be in a state of thorough preparation. With an army in the field, social difficulties, however arising or of whatever kind, speedily melt away and disappear under the influence of comradeship, of common dangers, and of common achievements, and the only thing then required of an army surgeon is that he should know his work and should do it thoroughly. But this requirement, in the changed conditions of modern warfare, can only be fulfilled by the aid of skilful organisation as well as of skilful individuals; and the persistent demand of the doctors for army rank is due to their recognition of this fact and to nothing else. The chief medical officer of a corps or of an army cannot deal effectively with the results of an engagement unless his position in relation to the general staff be such as to give him knowledge of the localities in which the services of his department are most likely to be required, nor unless his substantive rank as an army officer afford him the means of concentrating or otherwise disposing of his forces, that is to say, of the medical officers subordinate to him, of the officers and men of the bearer corps, of the ambulances, drivers, field hospitals, and hospital material, in such a way that they may be available when and where they are wanted. In the old days of regimental surgeons, such questions as these did not present themselves for solution. The surgeons and assistant surgeons accompanied their regiment, and were with it in the field. That system is the best possible in time of peace, but it is not calculated to stand the pressure of war, and it is dead beyond the possibility of revivification. What the red-tape army man fails to see is that changed conditions demand changed arrangements; and that military rank, only to be expressed by military titles, is essential to the government of bodies of men in the field. If the chief surgeon is to clear the front of wounded, he must have authority of the same sort as that which would enable a combatant officer to take the men under his command into action.

